# The Dwarf Diplomat

**Published:** 1881-07

**Authors:** 


### THE DWARF DIPLOMAT.

There is a dwarf in Paris who deserves a place among the historic pigmies the *Dispatch* lately alluded to. He is, indeed, a very remarkable figure in modern European history.

He is, in person, shorter than Tom Thumb by half an inch, an intelligent looking, ready witted, keen-eyed, well dressed and liberal little man, who drives in the Bois, in a carriage like a goat cart, drawn by a pony scarcely bigger than a dog, and smoking a cigar which rivals one of his arms in size. He lives in a handsome house near the Arc de Triomphe, with servants who stand in great awe of him. For little as there is of him, that little is very great.

When Isabella came to the throne she undertook to perpetuate several old customs of the Spanish court, that of the court dwarf among the number. Where she got this one from nobody knows. But he was a pigmy of keen wit, and shrewd good sense, and soon became a great favorite with her. She was not long in discovering that he had a very pretty talent for politics. Thenceforth he was her agent in all the plottings in which she was engaged. He was employed by Isabella in all sorts of confidential missions, and his assistance was invaluable in the cabals which set Alfonso on the throne. Nevertheless, Alfonso was ungrateful enough to leave him in Paris when he went to the throne the dwarf had helped provide for him, and when Isabella returned she was only permitted to do so on condition of leaving the dwarf behind. The fact was Alfonso was ashamed of him.

NEVER unsex yourself for greatness. Don't trample on flowers while longing for the stars. Live up to the full measure of life, give way to your impulses, loves and enthusiasms; sing, smile, labor and be happy.

